# A network-based approach for predicting key enzymes explaining metabolite abundance alterations in a disease phenotype

**DOI:** 10.1186/1752-0509-7-62

**Published:** 2013-07-19

**Authors:** Jon Pey, Luis Tobalina, Joaquín Prada J de Cisneros, Francisco J Planes

**Affiliations:** 1 CEIT and TECNUN, University of Navarra, Manuel de Lardizabal 15, San Sebastian 20018, Spain; 2 Institute of Infection, Immunity and Inflammation, University of Glasgow, Garscube Campus, Bearsden Road, Glasgow G61 1QH, Scotland

## Abstract

**Background:**

The study of metabolism has attracted much attention during the last years due to its relevance in various diseases. The advance in metabolomics platforms allows us to detect an increasing number of metabolites in abnormal high/low concentration in a disease phenotype. Finding a mechanistic interpretation for these alterations is important to understand pathophysiological processes, however it is not an easy task. The availability of genome scale metabolic networks and Systems Biology techniques open new avenues to address this question.

**Results:**

In this article we present a novel mathematical framework to find enzymes whose malfunction explains the accumulation/depletion of a given metabolite in a disease phenotype. Our approach is based on a recently introduced pathway concept termed Carbon Flux Paths (CFPs), which extends classical topological definition by including network stoichiometry. Using CFPs, we determine the Connectivity Curve of an altered metabolite, which allows us to quantify changes in its pathway structure when a certain enzyme is removed. The influence of enzyme removal is then ranked and used to explain the accumulation/depletion of such metabolite. For illustration, we center our study in the accumulation of two metabolites (*L-Cystine* and *Homocysteine*) found in high concentration in the brain of patients with mental disorders. Our results were discussed based on literature and found a good agreement with previously reported mechanisms. In addition, we hypothesize a novel role of several enzymes for the accumulation of these metabolites, which opens new strategies to understand the metabolic processes underlying these diseases.

**Conclusions:**

With personalized medicine on the horizon, metabolomic platforms are providing us with a vast amount of experimental data for a number of complex diseases. Our approach provides a novel apparatus to rationally investigate and understand metabolite alterations under disease phenotypes. This work contributes to the development of Systems Medicine, whose objective is to answer clinical questions based on theoretical methods and high-throughput “omics” data.

## Background

Metabolism comprises the inter-conversion of small molecules (metabolites) through enzymatically catalyzed biochemical reactions. These metabolites play a key role in different cellular functions, ranging from energy production to biosynthesis of complex macro-molecules. Metabolic alterations have been reported in a number of multifactorial diseases [1]. In particular, their abnormal role in cancer cells currently constitutes a hot topic in the field of molecular systems biology [2]. Several important works have recently emphasized this feature of cancer cells and have thrown light on their underlying complex regulatory processes [3], indicating novel ways to target malignant tumors. Extending the study of metabolic processes to other diseases is essential to complete our understanding of their key pathophysiological processes.

For this purpose, it is of utmost importance to exploit the information given by the spearhead experimental technologies that directly or indirectly provide a metabolic picture of different human samples. Gene expression analysis [4], quantitative protein measurement [5], metabolomics [6] and isotope labeling experiments [7] are the most widespread techniques when analyzing metabolic processes. Their integration into different mathematical models, mainly based on linear and non-linear optimization, has already provided relevant insights into different disease phenotypes [8,9]. Continuing this integration task is currently a relevant area in systems biology and medicine [10].

**Figure 1:**
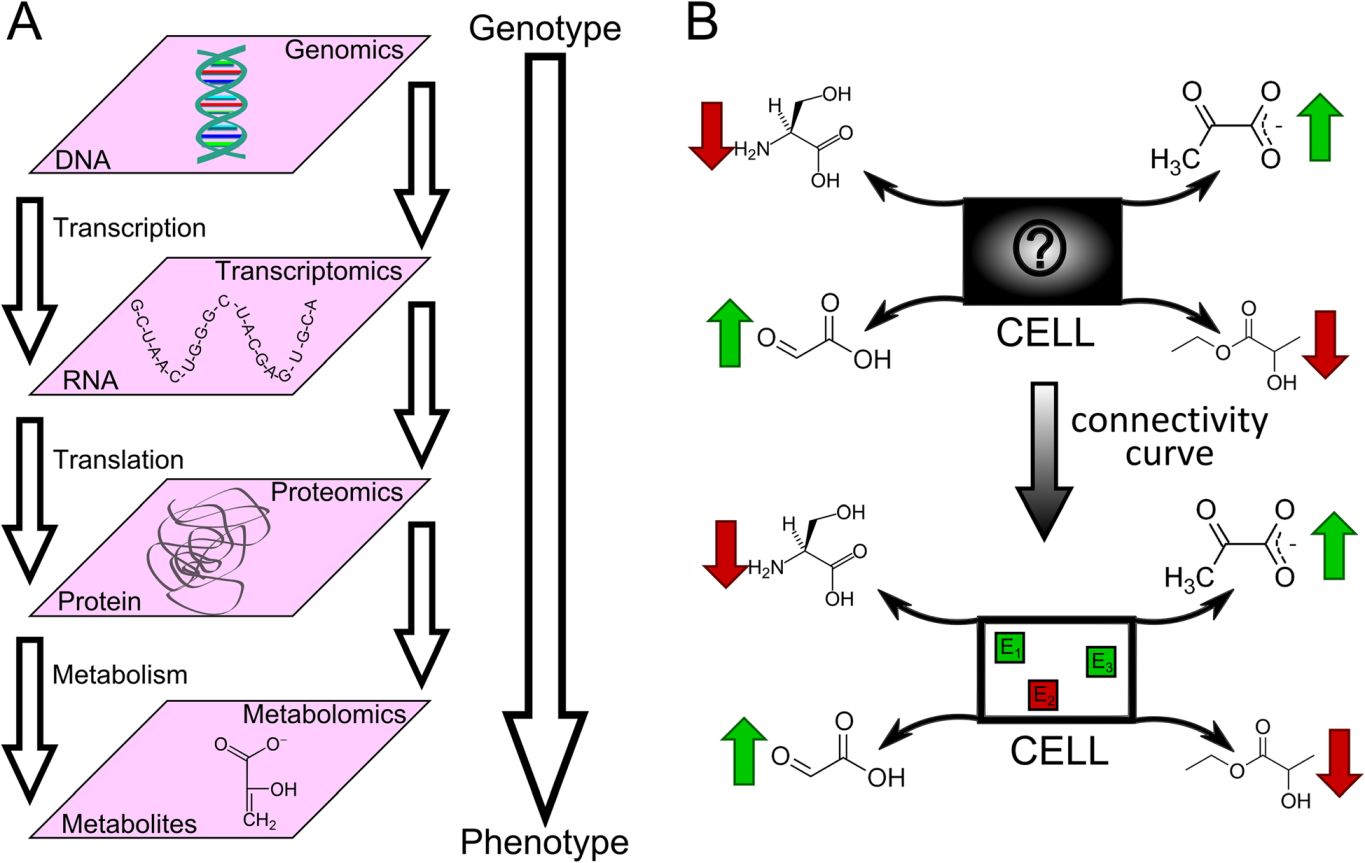
Schematic representation of A) different levels of complexity at molecular level; B) the purpose of our methodology.

The field of metabolomics has experienced a remarkable advance in mass spectrometry techniques and currently can measure hundreds of metabolites simultaneously [11]. In contrast with gene and protein expression, which are subject to stringent regulatory processes, metabolite abundance is closer to biochemical activity and therefore easier to correlate with cellular phenotype [12], as summarized in Figure 1A. For this reason, metabolomics has become a powerful approach for clinical diagnostics and personalized medicine [13]. In addition, metabolomics data potentially involves rich and valuable information to understand metabolic alterations underlying a disease phenotype. However, the detailed mechanistic interpretation of changes in metabolite abundance is not straightforward, as they may arise from different sources, some of them unlikely to be related with the phenotype of interest. Therefore, establishing effective methods to provide a functional interpretation to metabolomics data is required.

Assorted methods can be found in the literature to interpret metabolomics data based on curated metabolic pathways and networks [14]. On the one hand, kinetic [15] and thermodynamic [16] approaches directly relate metabolite concentrations with the activity of enzymes in a particular metabolic pathway. However, due to the high computational cost and intensive prior knowledge (kinetic constants/proteomics data) required by these methods, they have been directly applied to a limited number of metabolic pathways [17]. On the other hand, several tools mapping identified metabolites onto pre-defined metabolic pathways have been developed [18]. However, as pre-defined pathways do not fully capture complex metabolic states, it is difficult to extract the mechanism(s) responsible for changes in metabolite levels. To overcome this issue, different tools using network-based pathway (mathematical) definitions were introduced [19,20]. These methods take advantage of the plasticity and connectivity of genome-scale metabolic networks to provide a more compact functional view of metabolomics data [21].

In this article, we address the question as to find the key enzymes regarding the accumulation/depletion of identified metabolites (see Figure 1B). Given that metabolite abundance is recognized as the most accurate indicator of phenotype, deciphering enzymes that regulate such phenotype certainly constitutes a relevant question and links metabolite data with upstream molecular mechanisms. A similar investigation was conducted by Cakir et al. [22], where enzymes with statistically significant coordinated changes in the abundance of surrounding metabolites were identified. Given the high inter-connectivity of metabolic networks, key enzymes are not necessarily topological neighbors of identified metabolites, as assumed in that work. Instead, enzymes at considerable distances from identified metabolites may exert control on the flux of their underlying biosynthesis and degradation pathways and therefore alter their concentration.

In this light, we introduce a novel theoretical concept termed “connectivity curve (CC)”, which summarizes the structure of pathways consuming (producing) an identified metabolite and their underlying distances in a genome-scale metabolic network. Pathway distances are used as a clue for their fluxes in line with several theoretical works that show that shorter pathways support a higher flux than longer pathways [23-25]. Using CCs we examine whether the removal of a certain enzyme increases degradation (biosynthesis) pathway distances of an identified metabolite, which therefore would decrease its degradation (biosynthesis) fluxes and thus may lead to its accumulation (depletion).

In order to determine metabolic pathways and distances in CCs, we used Carbon Flux Paths (CFPs), a network-based pathway concept recently introduced by Pey et al. [26]. The CFP approach searches for simple paths (in the graph theoretical sense) between a given pair of source/target metabolites. In addition, the CFP approach ensures that the obtained path satisfies a relevant set of biophysical constraints, such as mass balance (usually referred to as steady-state condition), going beyond classical path finding approaches.

Based on CCs, we rank enzymes in the network as responsible for the accumulation (depletion) of an identified metabolite. To assess the performance of our approach, we investigated key enzymes corresponding to the accumulation of two metabolites (*L-Cysteine* and *Homocysteine*) closely related with mental disorders.

## Results and discussion

This section is organized as follows. We first illustrate the concept of CCs and several parameters arising from them by means of a classical metabolic network representing glucose metabolism [27]. This example is also used to describe the statistical validation conducted. We then discuss the resulting key enzymes obtained when applied our approach to explain the accumulation of *L-Cysteine* and *Homocysteine* in mental disorders.

### Connectivity Curve (CC) approach

Assume a metabolite is identified in significantly high concentration and we are concerned with finding enzymes whose malfunction explains its accumulation. For this purpose, we introduce the connectivity curve (CC), which plots the number of metabolites connected after moving away *n* steps (reactions) from such identified metabolite. In order to determine whether this identified metabolite is connected to other metabolites and their distances, we used the Carbon Flux Paths (CFPs) approach. The CFP algorithm searches for the shortest path between a pair of source and target metabolites in the context of the metabolic network, which is here represented as a metabolite-metabolite directed graph. To avoid not biologically meaningful shortcuts, we removed arcs not involving an effective carbon exchange. The use of integer linear programming allows us to impose further constraints. In particular, we incorporate reaction stoichiometry and force paths to satisfy the mass balance equation and perform in sustained steady-state. Further details can be found in Methods section.

**Figure 2:**
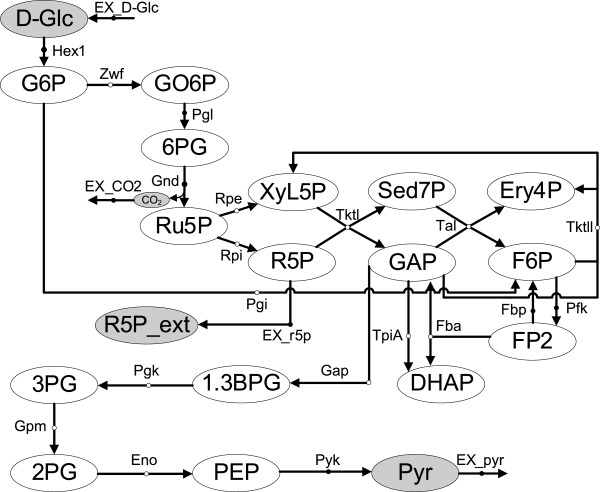
**Example metabolic network involving glycolysis and pentose phosphate pathway **[27]**.** Reaction arcs with a white circle indicate the reaction is reversible. Abbreviations of enzymes: Eno, enolase; Fba, fructose 1,6-bisphosphate aldolase; Fbp, fructose 1,6-bisphosphatase; Gap, glyceraldehydes 3-phosphate dehydrogenase; Gnd, phosphogluconate dehydrogenase (decarboxylating); Gpm, phosphoglycerate mutase; Hex1, hexokinase; Pfk, 6-phosphofructokinase; Pgi, phosphoglucoisomerase; Pgk, phosphoglycerate kinase; Pgl, phosphogluconolactonase; Pyk, pyruvate kinase; Rpe, ribulose-phosphate 3-epimerase; Rpi, ribose 5-phosphate isomerase; Tal, transaldolase; TktI, transketolase; TktII, transketolase; TpiA, triosephosphate isomerase; Zwf, glucose 6-phosphate dehydrogenase. Abbreviation of metabolites: 1,3BPG, 3-Phospho-D-glyceroyl phosphate; 2PG, D-Glycerate 2-phosphate; 3PG, 3-Phospho-D-glycerate; 6PG, 6-Phospho-D-gluconate; D-Glc, D-Glucose; DHAP, Dihydroxyacetone phosphate; Ery4P, D-Erythrose 4-phosphate; F6P, D-Fructose 6-phosphate; FP2, D-Fructose 1,6-bisphosphate; G6P, D-Glucose 6-phosphate; GAP, Glyceraldehyde 3-phosphate; GO6P, 6-phospho-D-glucono-1,5-lactone; PEP, phosphoenolpyruvate; Pyr, pyruvate; R5P, alpha-D-Ribose 5-phosphate; Ru5P, D-Ribulose 5-phosphate; Sed7P, sedoheptulose 7-phosphate; Xyl5P, D-Xylulose 5-phosphate.

**Figure 3:**
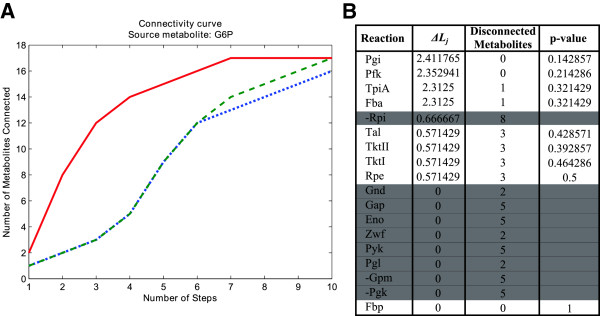
**Results arising from the example network in Figure**2**. A)** Connectivity curve for G6P in different scenarios; **B)** Length increasing parameter (∆*Lj*), disconnected metabolites and p-value for each enzyme knockout. Shaded reactions do not satisfy filtering criterion.

To illustrate the concept of CCs, consider the example metabolic network in Figure 2 based on Schuster et al. [27]. This network comprises the major part of the monosaccharide metabolism, involving glycolysis, pentose phosphate pathway (PPP) and part of gluconeogenesis, as well as an input flux of *Glucose* (D-Glc) and three output fluxes of CO_2_, *ribose-5-phosphate* (R5P) and *Pyruvate* (Pyr). We assumed that *Glucose-6-Phosphate* (G6P) is identified in high concentration and therefore enzymes responsible for its accumulation are evaluated. Based on CFPs, we calculated the minimum number of steps (reactions) necessary to reach any metabolite from G6P, *e.g.* 7 steps are required to reach Pyr. Note that the CFP approach is applied once for each metabolite different to G6P. Red solid line in Figure 3A shows the CC for G6P. We can observe that from G6P 2 metabolites can be reached in 1 step; 8 metabolites in 2 steps, etc.; finally reaching 17 metabolites in 7 steps. Full details can be found in the Additional file 1.

We assume that distances (number of steps) provide a clue of the degradation fluxes (velocity) of the metabolite under study, namely shorter distances imply higher fluxes. A more efficient use of resources via shorter pathways overall allows reactions to carry higher fluxes, which indirectly increases the capacity to produce biomass and energy. In particular, shorter pathways reduce mass leaks, wasted energy and the amount of protein required to catalyze a process, as discussed in a number of theoretical works [23-25]. There is also experimental evidence in *E. coli* evolution studies that the decrease in the number of active reaction steps and increase in growth rate occur simultaneously [28].

Based on the above, CCs can be used to evaluate changes in CFP distances in different scenarios. We particularly focus here on single reaction knockouts, *i.e.* we remove one-by-one each reaction from the metabolic network and re-calculate the CC for the identified metabolite. To illustrate this, green dashed line in Figure 3A shows the CC for G6P when *phosphoglucoisomerase* (Pgi) was knocked out. We can observe now that from G6P 1 metabolite can be reached in 1 step; 2 metabolites in 2 steps; 3 metabolites in 3 steps, etc.; finally reaching 17 metabolites in 10 steps. It is clear that the knockout of Pgi causes an increase in the distances from G6P to the rest of the metabolic network. The effect of this knockout in the set of CFPs from G6P to the rest of metabolites can be found in the Additional file 1. We repeat the procedure for each enzyme in the example network.

We hypothesize that the distance increase observed in our CCs approach after knocking-out a particular enzyme represents a decrease in the degradation flux of our identified metabolite, which may lead to its accumulation. As noted above, the basis is that shorter paths carry higher flux [23-25] and thus their blockage would lead to the usage of less efficient alternative pathways with the corresponding concentration alterations. Therefore, the malfunction of such enzymes may explain a significant increase in the concentration of the metabolite under study. Since we assume that the identified metabolite is closely related with a certain disease phenotype, these enzymes may constitute regulatory key points for such phenotype.

In order to quantitatively measure the increase in distances from the metabolite under study to the rest of the metabolic network when enzyme *j* is knocked out, we introduce the length increasing parameter (*∆L*_*j*_), which essentially averages distance differences between the knockout and wild-type scenarios (see Methods section). Note that we may have the case that, when an enzyme is knocked out, a number of metabolites become disconnected from the metabolite under study. In order to determine *∆L*_*j*_, we only consider changes in the distance to metabolites that remain connected after the knockout. To illustrate this consider the blue dotted line in Figure 3A, which represents the CC for G6P when *triosephosphate isomerase* (TpiA) was knocked out. We can observe that 16 metabolites are now reached from G6P, namely one less than in the wild-type scenario. In particular, *dihydroxyacetone phosphate* (DHAP) cannot be reached from G6P since TpiA is essential for its mass balance. Although we can still find routes linking G6P and DHAP, none of them can be mass balanced (see Additional file 1).

This parameter (*∆L*_*j*_) allows us to rank enzymes as responsible for the accumulation of the metabolite under consideration. Clearly, we are interested in enzymes that when knocked out present a high *∆L*_*j*_, which can potentially explain the accumulation of the metabolite of interest. Figure 3B details *∆L*_*j*_ in our example metabolic network. Pgi constitutes the most relevant enzyme for the accumulation of G6P. Indeed, blocking Pgi eliminates the classical pathways for glucose consumption, but others are still available. This can be easily observed in the analysis of Elementary Flux Modes (EFMs) conducted in the seminal work of Schuster et al. [27], namely few EFMs consuming G6P are left active after the knockout of Pgi and they involve more steps, for example, to reach Pyr. Note that we extract similar conclusions as when using the EFMs analysis because our CFP approach forces the mass balance constraint in the resulting paths. However, the use of EFMs approach for large metabolic networks is difficult due to combinatorial explosion [29]. We show below that our approach scales well even in such large metabolic networks.

We are aware of the fact that other mechanisms may lead to an accumulation or depletion of metabolites. For example, up-regulation of biosynthetic pathways of the metabolite under study may explain its accumulation. Note however that this is more difficult, since an increase in pathway flux is typically achieved if all enzymes [30] or (at least) a considerable number of enzymes in a pathway [31,32] are over-expressed. For simplicity, this strategy has been not considered in this work.

We would like to clarify that we have focused on the accumulation of identified metabolites, but the analysis of depleted metabolites can be easily accomplished. Indeed, the definition of CCs would slightly change, involving the number of metabolites reaching the metabolite under consideration after moving back *n* steps. Similarly, distances in CC provide a clue as to the biosynthesis fluxes of the identified metabolite. Therefore, when studying a depletion instead of an accumulation, we would search for enzymes that, when knocked-out, significantly increase such distances. This would decrease the biosynthesis flux of the metabolite under study and therefore lead to its depletion.

### Other issues: filtering criterion and specificity

As noted above, the knockout of certain enzymes may bring about the disconnection of pairs of metabolites. Clearly, if key metabolites are disconnected, important damage in cellular functions may be caused, even leading to cellular death. As we assume that phenotypic changes in metabolite abundance are more subtle, we are not aiming here at enzyme knockouts producing radical disconnections. Instead, we search for knockouts impairing but not disrupting the normal functioning of metabolic processes. For this purpose, we only consider enzymes that when knocked out do not alter the connectivity between the inputs (medium metabolites) and outputs (excreted metabolites) of the metabolic network. Note that we could impose other criteria, *e.g.* guaranteeing a particular biological function such as biomass production. To illustrate this, in the example in Figure 2 we have as inputs D-Glc and as outputs Pyr, CO_2_ and R5P. It can be easily observed that the knockout of *pyruvate kinase* (Pyk) disconnects the biosynthesis of Pyr from D-Glc, which violates our rule above and therefore it is not considered further. Instead, when TpiA is knocked out, although DHAP is disconnected from G6P, the connectivity between D-Glc and Pyr, CO_2_ and R5P is still found and therefore it is a viable knockout. Rows in the table in Figure 3B corresponding to enzymes violating this rule are shaded in grey. To conduct this task, we again used the CFPs approach.

For the sake of simplicity, it would be preferable to relate each enzyme with a unique metabolite accumulation, *i.e.* knocking out a certain enzyme would only lead to significant changes in distances from a unique metabolite. To evaluate this, we introduce a parameter representing the specificity of the actual enzyme knockout for the accumulation of the metabolite under study. In particular, we assigned a p-value for each enzyme, which defines the probability of finding such enzyme in an equal/better position in the ranking of a different metabolite. Details can be found in Methods section. Figure 3B includes the p-values for each enzyme in our example metabolic network. Note here that the best p-value that can be attained in our approach is one over the number of metabolites in the network under study. Given the reduced number of metabolites involved in this example network, this explains why p-values are not particularly low. As can be seen in the next section, this lack of statistical power is overcome as the network size increases. The lowest p-value is found for Pgi, which indicates that its knockout leads to the accumulation of a reduced set of metabolites, clearly including G6P. As noted above, we selected the most parsimonious solution and focused on specific knockouts, *i.e.* those having a small p-value. But a high p-value might not be undesired for other questions, since complex diseases may potentially present alterations in the concentration of more than one metabolite.

### Case studies: metabolite accumulation in mental disorders

In this sub-section we apply the approach presented above to rank enzymes responsible for the accumulation of *L-Cysteine* (LCystin) and *Homocysteine* (HCys), metabolites found in high concentration in some mental disorders. For this purpose, we used the human metabolic network Recon1 [33]. As others methods from constraint-based modeling, the application of our approach is dependent on the definition of the growth medium, *i.e.* available substrates. In this work a minimal medium based on glucose and amino acids was used [34]. We allow a way out of the network for exchange (external) metabolites found in Recon1 not included in the growth medium.

As noted above, to avoid not meaningful shortcuts when applying the CFP method, we removed arcs not involving an effective carbon exchange in each reaction. Based on Recon1, a list of pairs of metabolites exchanging carbon atoms for each reaction was built (see Additional file 2). We also neglected carbon arcs corresponding to hub metabolites, namely CoA, CO_2_, AMP, ATP and ADP, as typically done in others works [35]. In addition, given their participation in a high number of reactions, they can cause not meaningful shortcuts and disrupt carbon flux along the path. Note however that those metabolites were not removed from the stoichiometric matrix and therefore they must be mass balanced (see Methods section).

#### L-Cystine accumulation

The accumulation of LCystin is a relevant phenotype in Cystinosis, which may cause different tissue failure. In particular, brain atrophy was observed in this condition [36,37]. The principal cause of this accumulation is associated with the malfunction of the LCystin transport across lysosomal membrane [38].

Using the approach presented above, we explore alternative scenarios leading to LCystin accumulation in the context of brain damage. Table 1 summarizes results arising from our approach (full results can be found in Additional file 3). In particular, we present details as to the four top-ranked enzymes responsible for the accumulation of LCystin. In order to evaluate the performance of our method, we discuss below the role of these enzymes in several mental disorders.

**Table 1:** Details of 4 top-ranked reactions responsible for the accumulation of LCystin

**Reaction names**	∆***L***_***j***_	**p-value**
Cysteine oxidase	0.700348	0.0060
Formaldehyde dehydrogenase	0.637731	0.0190
S-formylglutathione hydralase	0.637731	0.0190
Sulfite oxidase	0.636891	0.0060

The first enzyme in the ranking is *cysteine oxidase* (CYSO), also referred to as cysteine dioxigenase, which presents *∆L*_*j*_≈0.7. Perry et al. [39] reported a decreased activity of this enzyme in the brain of a Pantothenate kinase-associated neurodegeneration (PKAN) patient (disease formerly known as Hallervorden-Spatz syndrome). This syndrome is characterized by rigidity, spasticity, dystonia and dementia among others. In that work they also found an accumulation of LCystin in the globus pallidus of the brain, precisely the same region where they measured the decreased activity of CYSO, clearly in line with our hypothesis. It is relevant to note that we are aiming at enzyme malfunctions that could lead to LCystin accumulation and our first predicted enzyme turned out to have been previously related with this phenotype in the literature. It is worth to mention that the length of CFPs between LCystin and more than three hundred metabolites were affected by the removal of this enzyme. This fact makes infeasible to systematically arrive at a clear network that summarizes pathway changes for visual inspection, which emphasizes the advantages of using (numerical) parametric methods as the one introduced here.

In the second and third position appear *formaldehyde dehydrogenase* (FALDH) and *S-Formylglutathione hydralase* (SFGTH) both with *∆L*_*j*_=0.638. FALDH is currently split into two independent reactions: EC 4.4.1.22 *Glutathione-dependent formaldehyde-activating enzyme*, GFAE, and EC 1.1.1.284 *S-(hydroxymethyl) glutathione dehydrogenase*, ADH3 [40].

GFAE consumes *Formaldehyde*, a highly toxic metabolite previously reported in several mental disorders [41,42]. A known mechanism reducing this toxic metabolite is the formaldehyde oxidation pathway [43]. In essence, this pathway comprises the enzymes under study: GFAE, ADH3 and SFGTH. Thus, these three enzymes are of utmost importance to reduce *Formaldehyde* concentration. In light of this, some authors proposed to increase the activity of these *Formaldehyde*-consuming enzymes so as to decrease brain damage [42,44]. We suggest that this pathway protects brain against damaging processes by reducing not only *Formaldehyde* presence but also LCystin concentration. Note that FALDH and SFGTH have a greater p-value, which indicates that they are not as specific to the LCystin accumulation as CYSO. Hence, finding effects on other metabolites in the literature does not seem out of place.

The last enzyme included in Table 1 is *sulfite oxidase* (SULFOX) with *∆L*_*j*_=0.637. An insufficiency of this enzyme causes a disease known as sulfite oxidase deficiency, characterized by neurological disorders, mental retardation and brain degradation [45]. Dublin et al. [46], measured an accumulation of LCystin in urine in a patient with sulfite oxidase deficiency. However, the same study reported a depletion (not an accumulation) of this metabolite in blood. In any case, this study suggests a connection between LCystin and sulfite oxidase deficiency. Interestingly, note there is no carbon flux from LCystin to any metabolite taking part in SULFOX, since they are inorganic metabolites (*Sulfite*, *Sulfate*, H_2_O and H^+^) and cofactors (*Ferricytochrome c* and *Ferrocytochrome c*). However, this reaction is important to mass balance the obtained paths. When it is knocked out, original short paths become unavailable and the average distance increases. This remark highlights the importance of balancing the paths as previously claimed in Pey et al. [26].

#### Homocysteine accumulation

A similar study was conducted for the accumulation of HCys, which has been previously linked to Alzheimer's disease (AD) [47,48]. Full results when our approach was applied in this scenario can also be found in Additional file 3. The four top-ranked enzymes are shown in Table 2. A brief discussion as to the relevance of these enzymes in AD is presented below.

**Table 2:** Details of 4 top-ranked reactions responsible for the accumulation of HCys

**Reaction names**	∆***L***_***j***_	**p-values**
Phosphatidylethanolamine N-methyltransferase	0.768421	0.0010
S-Adenosyl-L-methionine reversible transport, mitochondrial	0.768421	0.0010
Phosphatidylserine decarboxylase	0.767333	0.0130
Phosphatidylserine flippase	0.767333	0.0130

The first enzyme in the ranking is *Phosphatidylethanolamine N-methyltransferase* (PEMT), with ∆L_j_=0.768. This mitochondrial enzyme catalyzes the methylation of *Phosphatidylethanolamine* (PE) producing *Phosphatidylcholine* (PC). This enzyme has been previously related with AD. In Johnson and Blusztajn [49], this enzyme is proposed as a possible target for AD. In particular, they suggest that activating PEMT would be beneficial for cholinergic neurons, since PC production would be promoted. A similar conclusion was achieved in the work of Guan et al. [50]. They localized a deficit of this enzyme in the frontal cortex of brain affected with AD, precisely one of its most affected regions. From a different angle, Selley [51], aims at the accumulation of *S-Adenosyl-L-homocysteine* (SAH) as a possible cause for the malfunction of this enzyme in liver for AD patients. Interestingly, in that work evidences are also found to relate the inhibition of PEMT and the accumulation of HCys, which is in line with our hypothesis.

*S-Adenosyl-L-methionine reversible transport* (SLC25A26) is found in the second position in Table 2, with *∆L*_*j*_=0.768. This enzyme transports mitochondrial SAH into cytosol and cytosolic *S-Adenosyl-L-methionine* (SAM) into mitochondria. It should be pointed out that this is the only mechanism producing mitochondrial SAM in the network under study [33]. As this metabolite is required for the activity of PEMT (first enzyme in the ranking), SLC25A26 is essential for PEMT, *i.e.* the lack of SLC25A26 inhibits PEMT since the latter cannot perform in sustained steady-state without the former. From a different perspective, note that the inhibition of SLC25A26 may lead to the accumulation of SAH in mitochondria since, to our knowledge, this enzyme is the only one consuming SAH in such compartment. Following the hypothesis presented in Selley [51], this would inhibit PEMT as mentioned in the previous discussion. In summary, from two different perspectives, one theoretical and another experimental, we highlight the importance of SLC25A26 to guarantee the activity of PEMT, which is closely related with an accumulation of HCys and AD [51].

Next enzyme appearing in Table 2 is *Phosphatidylserine decarboxylase* (PISD), with *∆L*_*j*_= 0.767. This mitochondrial enzyme decarboxylases *Phosphatidylserine* (Pser) producing a molecule of PE and CO_2_. A direct effect when the activity of PISD decays is the accumulation of PSer, which has been indicated as a molecular signature of AD patients [52]. In addition to this, Salvador et al. [53], provides insights of the decreased activity of PISD during aging, which is known to increase the risk of suffering AD.

The last enzyme appearing in Table 2 is *phosphatidylserine flippase* (PSFLIP), also with *∆L*_*j*_= 0.767. PSFLIP is an ATP-consuming transporter of Pser from the cytosol to the mitochondria. This enzyme helps to maintain the membrane lipid bilayer asymmetry. When asymmetric collapse occurs, a signaling mechanism of synaptosomal apoptosis is triggered [54], as it occurs in AD [55]. In Castegna et al. [54] it is proposed that the oxidative environment characteristic in AD might contain compounds that interfere with the activity of PSFLIP and this may produce the unwanted asymmetric collapse. Similar conclusions were presented by [56].

In conclusion, based on the literature, we found that the deficit of PEMT seems to have a direct connection with HCys accumulation in AD. Instead, the role of SLC25A26, PISD and PSFLIP is hypothetical. As SLC25A26 is essential for the activity of PEMT, its implication in HCys accumulation seems plausible, though additional experimental evidence is required. With respect to PISD and PSFLIP, we found an indirect association with HCys concentration through shared AD diagnosis. As AD is a complex disease, this link is not particularly compelling. Therefore, experimental work is required to validate the role of PISD and PSFLIP in HCys accumulation.

## Conclusions

In this work, we present a novel network-based framework to find candidate enzymes whose malfunction is responsible for the accumulation of a given metabolite. Our approach was applied to investigate the accumulation of *L-Cystine* (LCystin) and *Homocysteine* (HCys) in mental disorders. Results were then discussed based on literature and found a good agreement with previously reported mechanisms. In addition, we hypothesize a novel role of several enzymes for the accumulation of these metabolites, which opens new strategies to understand the metabolic processes underlying these diseases. This is illustrated, for example, with *S-Adenosyl-L-methionine reversible transport* (SLC25A26), whose relevance for the accumulation of HCys, to our knowledge, has not been previously reported.

Our in-silico framework is mainly founded on the study of Connectivity Curves (CCs) of the metabolite under study in different scenarios. In particular, CCs summarize the pathway structure from an identified metabolite and their underlying distances, which are used as a clue for their fluxes. For the metabolite under study, we evaluate changes in CCs when an enzyme *j* is removed via *∆L*_*j*_, namely based on the logic that an increase of distances in its degradation (biosynthesis) pathways potentially explains its accumulation (depletion). The central hypothesis here is that shorter pathways carry higher flux than longer pathways. This assumption is supported by several theoretical works and it seems plausible, particularly according to the results obtained. However, the integration of “omics” data into our approach, especially proteomics and gene expression data, constitutes a future research direction with the aim of providing a more realistic pathway activity.

In order to complement the ranking arising from CCs, we introduced a p-value for each enzyme, which is a quantitative parameter indicating the specificity of a particular enzyme knockout/malfunction to explain the metabolite alteration under consideration. For the sake of simplicity, we preferably focused on enzymes with higher specificity for the accumulation/depletion of the metabolite under study. However, a high p-value might not be undesired for other biological questions, since complex diseases may potentially present alterations in the concentration of more than one metabolite. This possibility will be explored in the future.

In addition, in our approach we did not consider enzyme knockouts disrupting key cellular metabolic functions. Despite the fact that metabolism is typically altered in a disease scenario, we assume that essential functions can be still accomplished in the absence of an enzyme. In other words, we are not seeking lethal knockouts but possible malfunctions explaining the observed accumulations/depletions. For this purpose, we only analyzed knockouts not producing disconnections between medium metabolites (substrates) and cellular excreted metabolites. Clearly, this criterion can be revisited and modified according to the biological scenario under study, e.g. forcing the production of a particular set of metabolites required for cellular growth.

In summary, our approach involves three main ingredients: CCs and their parameters, p-value and filtering criterion. These ingredients share the use of Carbon Flux Paths (CFPs) for their determination. This pathway concept was recently introduced and goes beyond path finding techniques by accounting for additional biophysical constraints. In order to apply the CFP to the human genome-scale metabolic network presented in Duarte et al. [33], we manually built a database indicating input and output metabolites that exchange carbon atoms in each of its reactions. This is now available for further research.

The effect of CFPs is particularly observed when our approach predicted the association of LCystin with the enzyme Sulfite Oxidase (SULFOX). By definition, CFPs involve carbon exchange in their intermediate reaction steps. However, there is no carbon flux from LCystin to any metabolite taking part in SULFOX, since they are inorganic metabolites and cofactors. Interestingly, this reaction is important to mass balance the obtained paths from LCystin to the rest of metabolites. This case also illustrates the idea that enzymes at considerable distance from the metabolite under consideration may regulate its concentration.

Given the relevance of CFPs in the performance of our approach, improving their accuracy is certainly relevant. As noted in Pey et al. [26], CFPs must still face different issues. In particular, guaranteeing carbon exchange between the source and target metabolites is essential and this is not fully satisfied in their current format. We ensure carbon exchange in each of its intermediate steps, but not between the source and target. In this direction, the release of databases incorporating atomic mapping of metabolites at large scale is promising [57].

In addition, our approach sacrificed some accuracy by neglecting classical regulatory mechanisms with the aim to extend our analysis to genome-scale metabolic networks. Regulatory information is certainly relevant for explaining changes in metabolite levels; however, it is scarce for genome-scale networks. The issue will require further consideration when such data becomes widely available.

Finally, we believe our approach will be a practical tool to study poorly understood disease phenotypes. Extending its application to other diseases (obesity, cancer, diabetes…) will be a major activity in the future, precisely with the emergence of metabolomics studies. This work contributes to the development of Systems Medicine, an emerging field aiming to provide answers to clinical questions based on theoretical methods and high-throughput “omics” data.

## Methods

### Carbon Flux Paths (CFPs)

In Pey et al. [26], we presented details as to the mathematical formulation of the CFP approach. Similarly to other graph-based methods for the analysis of metabolic networks, CFPs search for simple paths that link a pair of metabolites. However, CFPs satisfy two additional properties: i) ability to work in sustained steady-state; and ii) carbon exchange in each of its intermediate steps. The CFP approach is an integer linear program. We summarize below their main features.

$$\min\sum_{i=1}^{C}\sum_{j=1,j\neq i}^{C}u_{\mathrm{ij}}$$

subject to:

(1) $$\sum_{j=1}^{C}u_{\mathrm{\alpha j}}=\sum_{i=1}^{C}u_{\mathrm{i\beta}}=1$$

(2) $$\sum_{i=1}^{C}u_{\mathrm{j\alpha}}=\sum_{j=1}^{C}u_{\mathrm{\beta j}}=0$$

(3) $$\sum_{i=1}^{C}u_{\mathrm{ik}}=\sum_{j=1}^{C}u_{\mathrm{kj}}\;k=1,...,C;k\neq \alpha ,\beta$$

(4) $$\sum_{i=1}^{C}u_{\mathrm{ik}}\leq 1\;k=1,...,C$$

(5) $$\sum_{r=1}^{R}S_{\mathrm{cr}}v_{r}=0\;c=1,...,C$$

(6) $$z_{r}\leq v_{r}\leq Mz_{r}\;r=1,...,R$$

(7) $$z_{\lambda }+z_{\mu }\leq 1\;\forall \left(\lambda ,\mu \right)\in B$$

(8) $$\sum_{r=1,d_{\mathrm{ijr}}=1}^{R}z_{r}\geq u_{\mathrm{ij}}\;i=1,...,C;j=1,...,C;i\neq j$$

$$v_{r}\geq 0\;r=1,...,R$$

$$z_{r}\in \left\{0,1\right\}\;r=1,...,R$$

$$u_{\mathrm{ij}}\in \left\{0,1\right\}\;i=1,...,C;j=1,...,C;i\neq j$$

A CFP constitutes a steady-state (balanced) flux distribution that involves a directed path (in the graph theoretical sense) between a given source (α) and target (β) metabolites. We first consider variables and constraints to obtain a directed path between α and β. We then illustrate constraints for steady-state flux distributions. Finally, both sets of constraints are linked.

Assume we have a metabolic network that comprises *R* reactions and *C* metabolites. Let *S*_*cr*_ be the stoichiometric coefficient associated with metabolite *c* in reaction *r*. We used a (directed) graph representation of the network where nodes are metabolites and arcs represent carbon exchange between substrates and products of reactions.

Binary variables *u*_*ij*_ stand for active arcs involved in the directed path between α and β, namely *u*_*ij*_ = 1 when an arc between metabolites *i* and *j* is active, *u*_*ij*_ = 0 otherwise. By means of Equations (1) and (2) we ensure that one arc leaves the source and one enters the target metabolite; and that no arc enters the source nor leaves the target. For intermediate metabolites in the path, the number of arcs entering them must be equal to the number of arcs leaving them, as imposed by Equation (3). Equation (4) ensures that a metabolite cannot be revisited in the path.

A steady-state flux distribution satisfies Equation (5). Note that the continuous variable *v*_*r*_ represents the flux through reaction *r (r=1,…,R)*, which by definition is non-negative. We also define a set of binary variables *z*_*r*_ closely related with flux variables, namely *z*_*r*_ = 1 if *v*_*r*_ > 0 and *z*_*r*_ = 0 if *v*_*r*_ = 0. We need Equation (6) to link *v*_*r*_ and *z*_*r*_ variables. M is a big scalar constant that sets an upper bound for reaction fluxes. Note that we split reversible reactions into two single irreversible steps. Therefore, we need to prevent a reaction and its reverse from appearing together in any steady-state flux distribution. This is modeled in Equation (7), where the set B={(λ,μ)| reaction λ and reaction μ are the reverse of each other}.

Equation (8) is introduced to link path constraints, Equations (1)-(4), and flux balancing constraints, Equations (5)-(7), which guarantees that if an arc is selected, at least one of the reactions *r* with an existing arc (carbon exchange) between metabolites *i* and *j* (*d*_*ijr*_ = 1) must carry flux. This last constraint ensures that the directed path found can perform in sustained steady-state, as claimed in Pey et al. [26].

Finally, we use an objective function that minimizes the number of arcs in the directed path, i.e. the shortest path between α and β. Further details can be found in Pey et al. [26].

### Average length increasing parameter ***(***∆*L*_*j*_)

The average length increasing parameter (*∆L*_*j*_) provides the difference in the average number of steps of metabolites connected to the metabolite under study between a knockout (*K*) scenario of enzyme *j* ($L_{j}^{K}$) and the wild-type (*W*) scenario ($L_{j}^{W}$), as observed in Equation (9). We detail below how to calculate this parameter.

(9) $$\Delta L_{j}=L_{j}^{K}-L_{j}^{W}$$

(10) $$D_{j}^{\max,K}=\arg\min_{x}\left(\left|C_{x}^{K,j}\right|=\left|C_{\infty }^{K,j}\right|\right)$$

(11) $$D^{\max,W}=\arg\min_{x}\left(\left|C_{x}^{W}\right|=\left|C_{\infty }^{W}\right|\right)$$

(12) $$L_{j}^{K}=\frac{\sum_{i=1}^{D_{j}^{\max,K}}\left(\left|C_{i}^{K,j}\right|-\left|C_{i-1}^{K,j}\right|\right)\infty i}{\left|C_{\infty }^{K,j}\right|}$$

(13) $$L_{j}^{W}=\frac{\sum_{i=1}^{D^{\max,W}}\left(\left|C_{i}^{W}\cap C_{\infty }^{K,j}\right|-\left|C_{i-1}^{W}\cap C_{\infty }^{K,j}\right|\right)\infty i}{\left|C_{\infty }^{K,j}\right|}$$

$C_{i}^{K,j}$ is the set of metabolites connected to the source metabolite in at most *i* steps when enzyme *j* is knocked out, while $C_{i}^{W}$ is the same value in the wild-type scenario; $\left|C_{i}^{K,j}\right|$ and $\left|C_{i}^{W}\right|$ are the cardinality of sets $C_{i}^{K,j}$ and $C_{i}^{W}$, respectively. $C_{\infty }^{K,j}$ is the set of metabolites connected to the source metabolite after moving ∞ steps away. In other words, $C_{\infty }^{K,j}$ represents the full set of metabolites connected to the source metabolite when enzyme *j* is knocked out, being $\left|C_{\infty }^{K,j}\right|$ its cardinality. $C_{\infty }^{W}$ and $\left|C_{\infty }^{W}\right|$ represent the same in the wild-type scenario.

As observed in Equation (10), $D_{j}^{\max,K}$ is the minimum distance required to reach the full set of metabolites connected the metabolite under study when enzyme *j* is knocked out ($C_{\infty }^{K,j}$); *D*^max,*W*^ represents the same value in the wild-type scenario (see Equation (11)).

$L_{j}^{K}$ and $L_{j}^{W}$ average distances among metabolites connected to the source metabolite in the knockout and wild-type scenarios, respectively. See Equations (12)-(13). Note here that, in order to compare both scenarios, we only considered those metabolites that remain connected when enzyme *j* is knocked out. For this reason, we used the intersection expression in Equation (13).

### Enzyme p-value calculation

The p-value defines the probability of obtaining an at least as extreme outcome according to a predefined Null Hypothesis (*H*_*0*_). Therefore, an adequate *H*_*0*_ must be defined in the first place. In our particular statistical test, *H*_*0*_ represents that the ranking of an enzyme is not determined by the accumulated metabolite under study. In other words, the resulting positions of the enzymes in the ranking derived from our approach are not specific of the metabolite under study.

Based on this *H*_*0*_ definition, an appropriate distribution function should be introduced. We define a set of positions *X*_*i*_ for a particular enzyme *j* regarding the accumulation of *N* different metabolites (*X*_*1,*_*X*_*2*_*… X*_*N*_). We assume that all the variables *X*_*i*_ come from the same theoretical distribution *F*. In addition, we introduce F*_N_ as the cumulative sampling distribution associated with the random sample set (*X*_*1,*_*X*_*2*_*… X*_*N*_). In essence, F*_N_ ascribes a probability equal to *1/N* to each of the sample observations. The formal representation of F*_N_ is presented in Equation (14). Note here that *x* represents a given position in the ranking of enzyme *j*.

(14) $$F_{N}^{*}\left(x\right)=\frac{1}{N}\sum_{i=1}^{N}I_{\left(-\infty ,x\right]}\left(X_{i}\right)\;\forall x\in R$$

Note that *I*_*A*_*(y)* is the indicator function corresponding to the set A:

(15) $$I_{A}\left(y\right)=\left\{\begin{array}{c} 1\;\mathrm{if}\;y\in A\\ 0\;\mathrm{if}\;y\notin A \end{array}\right)$$

Once F*_N_ is correctly defined, the calculation of the p-values corresponding to a determined ranking is straightforward. Assuming that enzyme *j* appears in the *k*-th position in a particular ranking, the corresponding p-value is calculated as follows:

(16) $$p_{\mathrm{value}}\left(k\right)=F_{N}^{*}\left(k\right)$$

In the case study Results section, we selected 1000 random metabolites and ranked enzymes based on the CC approach, *i.e. N*=1000. The position in the ranking for each enzyme in the different scenarios (1000 random metabolite accumulation) is used to build its empirical distribution (F*_N_). This is then used to determine the p-value associated with the enzymes top-ranked in the metabolites under study.

## Abbreviations

AD: Alzheimer's disease; ADH3: S-(hydrosymethyl) glutathione dehydrogenase; CC: Connectivity curve; CFP: Carbon flux path; CYSO: Cysteine oxidase; D-Glc: Glucose; DHAP: Dihydroxyacetone phosphate; EFM: Elementary flux mode; FALDH: Formaldehyde dehydrogenase; G6P: Glucose-6-Phosphate; GFAE: Glutathione-dependent formaldehyde-activating enzyme; HCys: Homocysteine; LCystin: L-Cysteine; PC: Phosphatidylcholine; PE: Phosphatidylethanolamine; PEMT: Phosphatidylethanolamine N-methyltransferase; Pgi: Phosphoglucoisomerase; PISD: Phosphatidylserine decarboxylase; PKAN: Pantothenate kinase-associated neurodegeneration; PPP: Pentose phosphate pathway; Pser: Phosphatidylserine; PSFLIP: Phosphatidylserine flippase; Pyk: Pyruvate kinase; Pyr: Pyruvate; R5P: Ribose-5-phosphate; SAH: S-Adenosyl-L-homocysteine; SAM: S-Adenosyl-L-methionine; SFGTH: S-Formylglutathione hydralase; SLC25A26: S-Adenosyl-L-methionine reversible transport; SULFOX: Sulfite oxidase; TpiA: Triosephosphate isomerase.

## Competing interests

The authors declare that they have no competing interests.

## Authors’ contributions

JP and FJP conceived the study; JP and LT implemented the method and performed the analyses; JPC developed the carbon exchange database; JP, LT and FJP developed the method and wrote the manuscript. All authors discussed the results and read, commented and approved the final manuscript.

## Supplementary Material

Additional file 1 Toy example full details. Click here for file

Additional file 2 Manually curated carbon arcs from Recon1. Click here for file

Additional file 3 Lcystin and Hcys analysis. Click here for file

## References

[B1] Iyer A Fairlie DP Brown L Lysine acetylation in obesity, diabetes and metabolic disease Immunol Cell Biol 2011 90 39 46 22083525 10.1038/icb.2011.99

[B2] Cairns RA Harris IS Mak TW Regulation of cancer cell metabolism Nat Rev Cancer 2011 11 85 95 21258394 10.1038/nrc2981

[B3] Vander Heiden MG Locasale JW Swanson KD Sharfi H Heffron GJ Amador-Noguez D Christofk HR Wagner G Rabinowitz JD Asara JM Cantley LC Evidence for an alternative glycolytic pathway in rapidly proliferating cells Science 2010 329 1492 1499 10.1126/science.1188015 20847263 PMC3030121

[B4] Irizarry R Hobbs B Collin F Exploration, normalization, and summaries of high density oligonucleotide array probe level data Biostatistics 2003 4 249 264 10.1093/biostatistics/4.2.249 12925520

[B5] Liu H Sadygov RG Yates JR A model for random sampling and estimation of relative protein abundance in shotgun proteomics Anal Chem 2004 76 4193 4201 10.1021/ac0498563 15253663

[B6] Spratlin JL Serkova NJ Eckhardt SG Clinical applications of metabolomics in oncology: a review Clin Cancer Res 2009 15 431 440 10.1158/1078-0432.CCR-08-1059 19147747 PMC2676437

[B7] Lee PW Wahjudi PN Xu J Go VL Tracer-based metabolomics: Concepts and practices Clin Biochem 2010 43 1269 1277 10.1016/j.clinbiochem.2010.07.027 20713038 PMC2952699

[B8] Agren R Bordel S Mardinoglu A Pornputtapong N Nookaew I Nielsen J Reconstruction of genome-scale active metabolic networks for 69 human cell types and 16 cancer types using INIT PLoS Comput Biol 2012 8 e1002518 10.1371/journal.pcbi.1002518 22615553 PMC3355067

[B9] Pey J Rubio A Theodoropoulos C Cascante M Planes FJ Integrating tracer-based metabolomics data and metabolic fluxes in a linear fashion via elementary carbon modes Metab Eng 2012 14 344 353 10.1016/j.ymben.2012.03.011 22487533

[B10] Joyce AR Palsson BO The model organism as a system: integrating “omics” data sets Nat Rev Mol Cell Biol 2006 7 198 210 10.1038/nrm1857 16496022

[B11] Smith CA Want EJ O’Maille G Abagyan R Siuzdak G XCMS: Processing mass spectrometry data for metabolite profiling using nonlinear peak alignment, matching, and identification Anal Chem 2006 78 779 787 10.1021/ac051437y 16448051

[B12] Patti GJ Yanes O Siuzdak G Innovation: metabolomics: the apogee of the omics trilogy Nat Rev Mol Cell Biol 2012 13 263 269 10.1038/nrm3314 22436749 PMC3682684

[B13] Van der Greef J Hankemeier T McBurney RN Metabolomics-based systems biology and personalized medicine: moving towards n = 1 clinical trials? Pharmacogenomics 2006 7 1087 1094 10.2217/14622416.7.7.1087 17054418

[B14] Heijnen JJ Approximative kinetic formats used in metabolic network modeling Biotechnol Bioeng 2005 91 534 545 10.1002/bit.20558 16003779

[B15] Smallbone K Simeonidis E Swainston N Mendes P Towards a genome-scale kinetic model of cellular metabolism BMC Syst Biol 2010 4 6 10.1186/1752-0509-4-6 20109182 PMC2829494

[B16] Henry CS Jankowski MD Broadbelt LJ Hatzimanikatis V Genome-Scale Thermodynamic Analysis of Escherichia coli Metabolism Biophys J 2006 90 1453 1461 10.1529/biophysj.105.071720 16299075 PMC1367295

[B17] Yizhak K Benyamini T Liebermeister W Ruppin E Shlomi T Integrating quantitative proteomics and metabolomics with a genome-scale metabolic network model Bioinformatics 2010 26 i255 i260 10.1093/bioinformatics/btq183 20529914 PMC2881368

[B18] Suhre K Schmitt-Kopplin P MassTRIX: mass translator into pathways Nucleic Acids Res 2008 36 suppl 2 W481 W484 18442993 10.1093/nar/gkn194 PMC2447776

[B19] Antonov AV Dietmann S Wong P Mewes HW TICL – a web tool for network-based interpretation of compound lists inferred by high-throughput metabolomics FEBS J 2009 276 2084 2094 10.1111/j.1742-4658.2009.06943.x 19292876

[B20] Cottret L Wildridge D Vinson F Barrett MP Charles H Sagot M-F Jourdan F MetExplore: a web server to link metabolomic experiments and genome-scale metabolic networks Nucleic Acids Res 2010 38 suppl 2 W132 W137 20444866 10.1093/nar/gkq312 PMC2896158

[B21] Jourdan F Cottret L Huc L Wildridge D Scheltema R Hillenweck A Barrett M Zalko D Watson D Debrauwer L Use of reconstituted metabolic networks to assist in metabolomic data visualization and mining Metabolomics 2010 6 312 321 LA – English 10.1007/s11306-009-0196-9 20526351 PMC2874485

[B22] Cakir T Patil KR Onsan ZI Ulgen KO Kirdar B Nielsen J Integration of metabolome data with metabolic networks reveals reporter reactions Mol Syst Biol 2006 2 50 17016516 10.1038/msb4100085 PMC1682015

[B23] Meléndez-Hevia E Waddell TG Montero F Optimization of metabolism: the evolution of metabolic pathways toward simplicity through the game of the pentose phosphate cycle J Theor Biol 1994 166 201 220 10.1006/jtbi.1994.1018

[B24] de Ponce León M Cancela H Acerenza L A strategy to calculate the patterns of nutrient consumption by microorganisms applying a two-level optimisation principle to reconstructed metabolic networks J Biol Phys 2008 34 73 90 10.1007/s10867-008-9067-2 19669494 PMC2577741

[B25] Pfeiffer T Bonhoeffer S Evolution of cross-feeding in microbial populations Am Nat 2004 163 E126 E135 10.1086/383593 15266392

[B26] Pey J Prada J Beasley J Planes F Path finding methods accounting for stoichiometry in metabolic networks Genome Biol 2011 12 R49 10.1186/gb-2011-12-5-r49 21619601 PMC3219972

[B27] Schuster S Fell DA Dandekar T A general definition of metabolic pathways useful for systematic organization and analysis of complex metabolic networks Nat Biotech 2000 18 326 332 10.1038/73786 10700151

[B28] Cooper VS Lenski RE The population genetics of ecological specialization in evolving Escherichia coli populations Nature 2000 407 736 739 10.1038/35037572 11048718

[B29] Klamt S Stelling J Combinatorial complexity of pathway analysis in metabolic networks Mol Biol Rep 2002 29 233 236 10.1023/A:1020390132244 12241063

[B30] Kacser H Acerenza L A universal method for achieving increases in metabolite production Eur J Biochem 1993 216 361 367 10.1111/j.1432-1033.1993.tb18153.x 8375376

[B31] Fell DA Thomas S Physiological control of metabolic flux: the requirement for multisite modulation Biochem J 1995 311 35 39 7575476 10.1042/bj3110035 PMC1136115

[B32] Niederberger P Prasad R Miozzari G Kacser H A strategy for increasing an in vivo flux by genetic manipulations. The tryptophan system of yeast Biochem J 1992 287 2 473 479 1445205 10.1042/bj2870473 PMC1133189

[B33] Duarte NC Becker SA Jamshidi N Thiele I Mo ML Vo TD Srivas R Palsson BØ Global reconstruction of the human metabolic network based on genomic and bibliomic data Proc Natl Acad Sci USA 2007 104 1777 1782 10.1073/pnas.0610772104 17267599 PMC1794290

[B34] Folger O Jerby L Frezza C Gottlieb E Ruppin E Shlomi T Predicting selective drug targets in cancer through metabolic networks Mol Syst Biol 2011 7 501 21694718 10.1038/msb.2011.35 PMC3159974

[B35] Planes FJ Beasley JE An optimization model for metabolic pathways Bioinformatics 2009 25 2723 2729 10.1093/bioinformatics/btp441 19620100

[B36] Cochat P Drachman R Gagnadoux MF Pariente D Broyer M Cerebral atrophy and nephropathic cystinosis Arch Dis Child 1986 61 401 403 10.1136/adc.61.4.401 3707194 PMC1777739

[B37] Feksa LR Cornelio A Dutra-Filho CS De Souza Wyse AT Wajner M Wannmacher CMD Inhibition of pyruvate kinase activity by cystine in brain cortex of rats Brain Res 2004 1012 93 100 10.1016/j.brainres.2004.03.035 15158165

[B38] Gahl WA Thoene JG Schneider JA Cystinosis N Engl J Med 2002 347 111 121 10.1056/NEJMra020552 12110740

[B39] Perry TL Norman MG Yong VW Whiting S Crichton JU Hansen S Kish SJ Hallervorden-Spatz disease: cysteine accumulation and cysteine dioxygenase deficiency in the globus pallidus Ann Neurol 1985 18 482 489 10.1002/ana.410180411 4073841

[B40] Carbon S Ireland A Mungall CJ Shu S Marshall B Lewis S AmiGO: online access to ontology and annotation data Bioinformatics 2009 25 288 289 10.1093/bioinformatics/btn615 19033274 PMC2639003

[B41] Perna RB Bordini EJ Deinzer-Lifrak M A case of claimed persistent neuropsychological sequelae of chronic formaldehyde exposure: clinical, psychometric, and functional findings Arch Clin Neuropsychol 2001 16 33 44 14590191

[B42] Tong Z Zhang J Luo W Wang W Li F Li H Luo H Lu J Zhou J Wan Y He R Urine formaldehyde level is inversely correlated to mini mental state examination scores in senile dementia Neurobiol Aging 2011 32 31 41 10.1016/j.neurobiolaging.2009.07.013 19879019

[B43] Romero P Wagg J Green M Kaiser D Krummenacker M Karp P Computational prediction of human metabolic pathways from the complete human genome Genome Biol 2004 6 R2 10.1186/gb-2004-6-1-r2 15642094 PMC549063

[B44] Mori O Haseba T Kameyama K Shimizu H Kudoh M Ohaki Y Arai Y Yamazaki M Asano G Histological distribution of class III alcohol dehydrogenase in human brain Brain Res 2000 852 186 190 10.1016/S0006-8993(99)02201-5 10661511

[B45] Kisker C Schindelin H Pacheco A Wehbi WA Garrett RM Rajagopalan KV Enemark JH Rees DC Molecular basis of sulfite oxidase deficiency from the structure of sulfite oxidase Cell 1997 91 973 983 10.1016/S0092-8674(00)80488-2 9428520

[B46] Dublin AB Hald JK Wootton-Gorges SL Isolated sulfite oxidase deficiency: MR imaging features Am J Neuroradiol 2002 23 484 485 11901024 PMC7975306

[B47] Ravaglia G Forti P Maioli F Martelli M Servadei L Brunetti N Porcellini E Licastro F Homocysteine and folate as risk factors for dementia and Alzheimer disease Am J Clin Nutr 2005 82 636 643 16155278 10.1093/ajcn.82.3.636

[B48] McCaddon A Davies G Hudson P Tandy S Cattell H Total serum homocysteine in senile dementia of Alzheimer type Int J Geriatr Psychiatry 1998 13 235 239 10.1002/(SICI)1099-1166(199804)13:4<235::AID-GPS761>3.0.CO;2-8 9646150

[B49] Johnson PI Blusztajn JK Sexually dimorphic activation of liver and brain phosphatidylethanolamine N-methyltransferase by dietary choline deficiency Neurochem Res 1998 23 583 587 10.1023/A:1022470301550 9566595

[B50] Guan Z Wang Y Xiao K Hu P Liu J Activity of phosphatidylethanolamine-N-methyltransferase in brain affected by Alzheimers disease Neurochem Int 1999 34 41 47 10.1016/S0197-0186(98)00068-0 10100195

[B51] Selley ML A metabolic link between S-adenosylhomocysteine and polyunsaturated fatty acid metabolism in Alzheimer’s disease Neurobiol Aging 2007 28 1834 1839 10.1016/j.neurobiolaging.2006.08.003 16996649

[B52] Ma K Langenbach R Rapoport SI Basselin M Altered brain lipid composition in cyclooxygenase-2 knockout mouse J Lipid Res 2007 48 848 854 10.1194/jlr.M600400-JLR200 17202128

[B53] Salvador GA López FM Giusto NM Age-related changes in central nervous system phosphatidylserine decarboxylase activity J Neurosci Res 2002 70 283 289 10.1002/jnr.10385 12391587

[B54] Castegna A Lauderback CM Mohmmad-Abdul H Butterfield DA Modulation of phospholipid asymmetry in synaptosomal membranes by the lipid peroxidation products, 4-hydroxynonenal and acrolein: implications for Alzheimer’s disease Brain Res 2004 1004 193 197 10.1016/j.brainres.2004.01.036 15033435

[B55] Bader Lange ML Cenini G Piroddi M Mohmmad Abdul H Sultana R Galli F Memo M Butterfield DA Loss of phospholipid asymmetry and elevated brain apoptotic protein levels in subjects with amnestic mild cognitive impairment and Alzheimer disease Neurobiol Dis 2008 29 456 464 10.1016/j.nbd.2007.11.004 18077176 PMC2292396

[B56] Butterfield DA Bader Lange ML Sultana R Involvements of the lipid peroxidation product, HNE, in the pathogenesis and progression of Alzheimer’s disease Biochimica et Biophysica Acta (BBA) - Molecular and Cell Biology of Lipids 2010 1801 924 929 10.1016/j.bbalip.2010.02.005 PMC2886168 20176130

[B57] Ravikirthi P Suthers PF Maranas CD Construction of an E. Coli genome-scale atom mapping model for MFA calculations Biotechnol Bioeng 2011 108 1372 1382 10.1002/bit.23070 21328316

