# Supplementary material for: A network-based approach for predicting key enzymes explaining metabolite abundance alterations in a disease phenotype
Source: BMC Syst Biol. 2013 Jul 19;7:62. doi: 10.1186/1752-0509-7-62 (PMC3733687; doi:10.1186/1752-0509-7-62)
Supplement: Additional file 1 — Toy example full details. [file 1752-0509-7-62-S1.docx]

**Additional file I. Toy example full details**

Jon Pey^1,†^, Luis Tobalina^1,†^, Joaquín Prada J. de Cisneros^1,2^, and Francisco J. Planes^1,*^

*^1^CEIT and Tecnun, University of Navarra, San Sebastian, Spain.*

*^2^ Institute of Infection, Immunity and Inflammation, University of Glasgow, Garscube Campus, Bearsden Road, Glasgow G61 1QH.*

^†^*Both authors have equally contributed to this work*

*^*^Corresponding authors: fplanes@ceit.es*

Example metabolic network in Figure 2 of the main manuscript is based on that presented in Schuster et al. [1]. The stoichiometry of the reactions was taken from the Human Metabolic Network Recon1 [2]. Details as to metabolites, reactions and carbon arcs are presented in tables S1, S2 and S3, respectively.

| **Metabolite** | **Full name** |
| --- | --- |
| H | *H+* |
| ATP | *ATP* |
| H2O | *H2O* |
| ADP | *ADP* |
| Pi | *Phosphate* |
| NAD | *Nicotinamide adenine dinucleotide* |
| NADH | *Nicotinamide adenine dinucleotide - reduced* |
| NADPH | *Nicotinamide adenine dinucleotide phosphate - reduced* |
| NADP | *Nicotinamide adenine dinucleotide phosphate* |
| CO2 | *CO2* |
| GAP | *Glyceraldehyde 3-phosphate* |
| 6PG | *6-Phospho-D-gluconate* |
| Ru5P | *D-Ribulose 5-phosphate* |
| xyl5P | *D-Xylulose 5-phosphate* |
| DHAP | *Dihydroxyacetone phosphate* |
| PEP | *Phosphoenolpyruvate* |
| Sed7P | *Sedoheptulose 7-phosphate* |
| Ery4P | *D-Erythrose 4-phosphate* |
| F6P | *D-Fructose 6-phosphate* |
| Pyr | *Pyruvate* |
| G6P | *D-Glucose 6-phosphate* |
| 1,3BPG | *3-Phospho-D-glyceroyl phosphate* |
| 3PG | *3-Phospho-D-glycerate* |
| 2PG | *D-Glycerate 2-phosphate* |
| FP2 | *D-Fructose 1,6-bisphosphate* |
| R5P | *alpha-D-Ribose 5-phosphate* |
| GO6P | *6-phospho-D-glucono-1,5-lactone* |
| R5P_ext | *alpha-D-Ribose 5-phosphate external* |
| D-Glc | *D-Glucose* |

**Table S1:** Details of metabolites involved in the toy example

| **Reaction** | **Full name** | **Formula** |
| --- | --- | --- |
| Gnd | *phosphogluconate dehydrogenase* | 6PG + NADP --> CO2 + NADPH + Ru5P |
| Tal | *transaldolase* | GAP + Sed7P <==> Ery4P + F6P |
| Pgi | *glucose-6-phosphate isomerase* | G6P <==> F6P |
| Gap | *glyceraldehyde-3-phosphate dehydrogenase* | GAP + NAD + Pi <==> 1,3BPG + H + NADH |
| Pfk | *phosphofructokinase* | ATP + F6P --> ADP + FP2 + H |
| Eno | *Enolase* | 2PG <==> H2O + PEP |
| Fbp | *fructose-bisphosphatase* | FP2 + H2O --> F6P + Pi |
| TpiA | *triose-phosphate isomerase* | DHAP <==> GAP |
| TktII | *transketolase* | Ery4P + Xyl5P <==> F6P + GAP |
| Gpm | *phosphoglycerate mutase* | 2PG <==> 3PG |
| TktI | *transketolase* | R5P + Xyl5P <==> GAP + Sed7P |
| Pgk | *phosphoglycerate kinase* | 3PG + ATP <==> 1,3BPG + ADP |
| Rpe | *ribulose 5-phosphate 3-epimerase* | Ru5P <==> Xyl5P |
| Rpi | *ribose-5-phosphate isomerase* | R5P <==> Ru5P |
| Zwf | *glucose 6-phosphate dehydrogenase* | G6P + NADP <==> GO6P + H + NADPH |
| Fba | *fructose-bisphosphate aldolase* | FP2 <==> DHAP + GAP |
| Pyk | *pyruvate kinase* | ADP + H + PEP --> ATP + Pyr |
| Pgl | *6-phosphogluconolactonase* | GO6P + H2O --> 6PG + H |
| EX_r5p |  | R5P --> R5P_ext |
| Hex1 | *hexokinase (D-glucose:ATP)* | ATP + D-Glc --> ADP + G6P + H |

**Table S2:** Details of reactions involved in the toy example

| **Source** | **Target** | **Reaction** |
| --- | --- | --- |
| NADP | NADPH | Gnd |
| 6PG | Ru5P | Gnd |
| GAP | F6P | Tal |
| Sed7P | Ery4P | Tal |
| Sed7P | F6P | Tal |
| G6P | F6P | Pgi |
| NAD | NADH | Gap |
| GAP | 1,3BPG | Gap |
| F6P | FP2 | Pfk |
| 2PG | PEP | Eno |
| FP2 | F6P | Fbp |
| DHAP | GAP | TpiA |
| xyl5P | GAP | TktII |
| xyl5P | F6P | TktII |
| Ery4P | F6P | TktII |
| 2PG | 3PG | Gpm |
| xyl5P | GAP | TktI |
| xyl5P | Sed7P | TktI |
| R5P | Sed7P | TktI |
| 3PG | 1,3BPG | Pgk |
| Ru5P | xyl5P | Rpe |
| R5P | Ru5P | Rpi |
| NADP | NADPH | Zwf |
| G6P | GO6P | Zwf |
| FP2 | GAP | Fba |
| FP2 | DHAP | Fba |
| PEP | Pyr | Pyk |
| GO6P | 6PG | Pgl |
| R5P | R5P[e] | Prs_DeoB |
| D-Glc | G6P | Hex1 |

**Table S3:** Details of carbon arcs involved in the toy example

For convenience, we also added exchange reactions to the list of metabolites in Table S4. In the work of Schuster et al. [1] these metabolites were considered external metabolites.

| **Metabolite** | **Full name** |
| --- | --- |
| H | *H+* |
| ATP | *ATP* |
| H2O | *H2O* |
| ADP | *ADP* |
| Pi | *Phosphate* |
| NAD | *Nicotinamide adenine dinucleotide* |
| NADH | *Nicotinamide adenine dinucleotide - reduced* |
| NADPH | *Nicotinamide adenine dinucleotide phosphate - reduced* |
| NADP | *Nicotinamide adenine dinucleotide phosphate* |
| CO2 | *CO2* |
| Pyr | *Pyruvate* |
| D-Glc | *D-Glucose* |
| R5P_ext | *alpha-D-Ribose 5-phosphate external* |

**Table S4:** Additional exchange reactions in the toy example

We assumed that *Glucose-6-Phosphate* (G6P) is identified in high concentration and therefore enzymes responsible for its accumulation are evaluated. As noted in the main paper, we search for enzymes whose knockout increase distances of degradation pathways of G6P and may therefore explain such accumulation. Aiming at guaranteeing an effective consumption of G6P and avoid recirculation via cyclic pathways when balancing the path, we added an exchange reaction for the metabolite under study (here G6P). In order to force this reaction, we included an artificial metabolite as substrate of this reaction, in our case G6P[a], as observed in Figure S1. Overall, we added the following reaction: G6P[a]🡪G6P, which logically involves a carbon arc exchange. Clearly, stoichiometric balance is not applied to the artificial metabolite.

**Finding paths from G6P[a] (instead of G6P) to the rest of metabolites guarantees effective consumption of G6P.** In terms of CFP distances, this step is not taken into account. For this reason we remove this step from resulting CFPs, as observed below. We provide below resulting CFPs in the different scenarios discussed in the main manuscript.

Finally, note that this technical detail was also used in the analysis of Lcystin and Hcys.


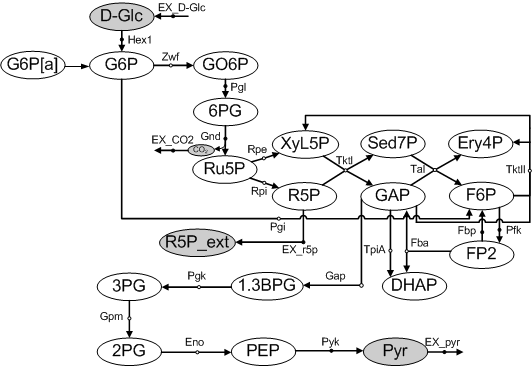


**Figure S1:** Example metabolic network including artificial reaction exchange for G6P

# CFPs obtained in Wild-Type:

G6P --> F6P --> GAP

G6P --> GO6P --> 6PG

G6P --> F6P --> xyl5P --> Ru5P

G6P --> F6P --> xyl5P

G6P --> F6P --> FP2 --> DHAP

G6P --> F6P --> GAP --> 1,3BPG --> 3PG --> 2PG --> PEP

G6P --> F6P --> Sed7P

G6P --> F6P --> Ery4P

G6P --> F6P

G6P --> F6P --> GAP --> 1,3BPG --> 3PG --> 2PG --> PEP --> Pyr

G6P --> F6P --> GAP --> 1,3BPG

G6P --> F6P --> GAP --> 1,3BPG --> 3PG

G6P --> F6P --> GAP --> 1,3BPG --> 3PG --> 2PG

G6P --> F6P --> FP2

G6P --> F6P --> Sed7P --> R5P

G6P --> GO6P

G6P --> F6P --> Sed7P --> R5P --> R5P[e]

# CFPs obtained with Pgi knocked-out:

G6P --> GO6P --> 6PG --> Ru5P --> xyl5P --> GAP

G6P --> GO6P --> 6PG

G6P --> GO6P --> 6PG --> Ru5P

G6P --> GO6P --> 6PG --> Ru5P --> xyl5P

G6P --> GO6P --> 6PG --> Ru5P --> xyl5P --> F6P --> FP2 --> DHAP

G6P --> GO6P --> 6PG --> Ru5P --> xyl5P --> GAP --> 1,3BPG --> 3PG --> 2PG --> PEP

G6P --> GO6P --> 6PG --> Ru5P --> xyl5P --> Sed7P

G6P --> GO6P --> 6PG --> Ru5P --> R5P --> Sed7P --> Ery4P

G6P --> GO6P --> 6PG --> Ru5P --> xyl5P --> F6P

G6P --> GO6P --> 6PG --> Ru5P --> xyl5P --> GAP --> 1,3BPG --> 3PG --> 2PG --> PEP --> Pyr

G6P --> GO6P --> 6PG --> Ru5P --> xyl5P --> GAP --> 1,3BPG

G6P --> GO6P --> 6PG --> Ru5P --> xyl5P --> GAP --> 1,3BPG --> 3PG

G6P --> GO6P --> 6PG --> Ru5P --> xyl5P --> GAP --> 1,3BPG --> 3PG --> 2PG

G6P --> GO6P --> 6PG --> Ru5P --> xyl5P --> F6P --> FP2

G6P --> GO6P --> 6PG --> Ru5P --> R5P

G6P --> GO6P

G6P --> GO6P --> 6PG --> Ru5P --> R5P --> R5P[e]

# CFPs obtained with TpiA knocked-out:

G6P --> GO6P --> 6PG --> Ru5P --> xyl5P --> GAP

G6P --> GO6P --> 6PG

G6P --> GO6P --> 6PG --> Ru5P

G6P --> GO6P --> 6PG --> Ru5P --> xyl5P

G6P --> GO6P --> 6PG --> Ru5P --> xyl5P --> GAP --> 1,3BPG --> 3PG --> 2PG --> PEP

G6P --> GO6P --> 6PG --> Ru5P --> R5P --> Sed7P

G6P --> GO6P --> 6PG --> Ru5P --> xyl5P --> Sed7P --> Ery4P

G6P --> GO6P --> 6PG --> Ru5P --> xyl5P --> F6P

G6P --> GO6P --> 6PG --> Ru5P --> xyl5P --> GAP --> 1,3BPG --> 3PG --> 2PG --> PEP --> Pyr

G6P --> GO6P --> 6PG --> Ru5P --> xyl5P --> GAP --> 1,3BPG

G6P --> GO6P --> 6PG --> Ru5P --> xyl5P --> GAP --> 1,3BPG --> 3PG

G6P --> GO6P --> 6PG --> Ru5P --> xyl5P --> GAP --> 1,3BPG --> 3PG --> 2PG

G6P --> GO6P --> 6PG --> Ru5P --> xyl5P --> F6P --> FP2

G6P --> GO6P --> 6PG --> Ru5P --> R5P

G6P --> GO6P

G6P --> GO6P --> 6PG --> Ru5P --> R5P --> R5P[e]

# References

1. Schuster S, Fell DA, Dandekar T: **A general definition of metabolic pathways useful for systematic organization and analysis of complex metabolic networks**. *Nat Biotech* 2000, **18**:326-332.

2. Duarte NC, Becker SA, Jamshidi N, Thiele I, Mo ML, Vo TD, Srivas R, Palsson BØ: **Global reconstruction of the human metabolic network based on genomic and bibliomic data**. *Proceedings of the National Academy of Sciences* 2007, **104**:1777-1782.
